# Safety and Efficacy of the ACE-Inhibitor Ramipril in Alport Syndrome: The Double-Blind, Randomized, Placebo-Controlled, Multicenter Phase III EARLY PRO-TECT Alport Trial in Pediatric Patients

**DOI:** 10.5402/2012/436046

**Published:** 2012-07-01

**Authors:** Oliver Gross, Tim Friede, Reinhard Hilgers, Anke Görlitz, Karsten Gavénis, Raees Ahmed, Ulrike Dürr

**Affiliations:** ^1^Department of Nephrology and Rheumatology, University Medical Center Göttingen, Robert-Koch Stra**β**e 40, 37075 Göttingen, Germany; ^2^Department of Medical Statistics, University Medical Center Göttingen, 37075 Göttingen, Germany; ^3^Institute for Applied Research and Clinical Trials GmbH (IFS) and Georg August University Göttingen, 37075 Göttingen, Germany

## Abstract

*Introduction*. Retrospective observational data show that ACE-inhibitor therapy delays renal failure and improves life expectancy in Alport patients with proteinuria. The EARLY PRO-TECT Alport trial assesses the safety and efficacy of early therapy onset with ramipril in pediatric Alport patients. *Methods and analysis*. This double-blind, randomized, placebo-controlled, multicenter phase III trial (NCT01485978; EudraCT-number 2010-024300-10) includes 120 pediatric patients aged 24 months to 18 years with early stages of Alport syndrome (isolated hematuria or microalbuminuria). From March 2012, up to 80 patients will be randomized 1:1 to ramipril or placebo. In the event of disease progression during 3-year treatment, patients are unblinded and ramipril is initiated, if applicable. Approximately 40 patients receive open-label ramipril contributing to the safety database. Primary end-points are “time to progression to next disease level” and “incidence of adverse drug events before disease progression.” Treatment effect estimates from the randomized comparison and Alport registry data will be combined in supportive analyses to maximize evidence. *Conclusion*. Without this trial, ACE inhibitors may become standard off-label treatment in Alport syndrome without satisfactory evidence base. The results are expected to be of relevance for therapy of all pediatric patients with kidney disease, and the trial protocol might serve as a model for other rare pediatric glomerulopathies.

## 1. Background

More than two decades have passed since the identification of COL4A5 gene as the genetic locus for X-linked Alport syndrome (AS) [1]. The hereditary type IV collagen disease Alport syndrome leads to progressive kidney fibrosis and end-stage renal failure. Knowledge of the mutation has allowed early genetic diagnosis [1] of AS years or even decades before renal failure. Still, despite early diagnosis, no evaluated therapy could be offered until recently.

Animal studies on AS, based on the fundamental genetic discoveries, have produced a variety of potentially effective therapies for Alport kidney disease [2–5]. In “Alport-mice,” early therapy onset with the angiotensin-converting-enzyme-inhibitor (ACEI) ramipril doubled the lifespan until renal failure [5]. This basic research results have led ACEIs to be the standard off-label therapy of AS for most nephrologists, including pediatric nephrologists. Retrospective observational data have shown that ACEIs reduce proteinuria [6] and, most important, delay end-stage renal failure and thus improve life expectancy in Alport patients [7]. The data show the efficacy of ACEI treatment of AS patients with proteinuria (and to a lesser extent in patients with ongoing renal failure). To date, no serious side effects of ACEIs have been reported to the North American or European Alport syndrome registries. However, retrospective registry data do not guarantee the comprehensive reporting of serious adverse events (SAEs) and adverse drug reactions (ADRs). ACEIs are not authorized for use in children with proteinuria or any type of renal disease [8, 9]. ACEIs are currently authorized for use in hypertensive children aged 8 years and older. No controled clinical trial has ever assessed the safety and efficacy of ACEIs in pediatric AS patients. Safety data from adults are poorly predictive for children. Thus, decisions on the off-label treatment of children with AS are currently made by the treating pediatrician.

The prospective, randomized EARLY PRO-TECT Alport trial was planned to provide valid data as the basis for a treatment recommendation and a subsequent marketing authorization for ramipril in children with AS. Without this trial, ACEIs may become the de facto standard off-label treatment of Alport syndrome without satisfactory evidence base.

## 2. Methods and Analysis

### 2.1. Design

**Figure 1:**
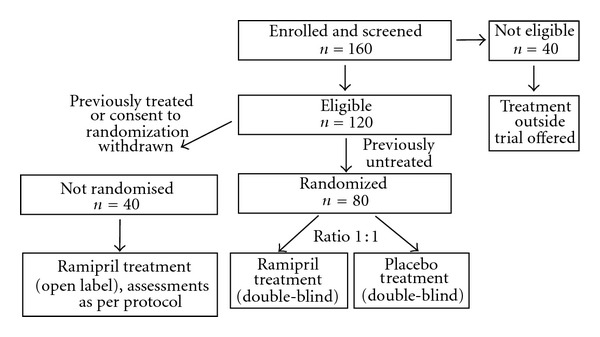
Study design of the EARLY PRO-TECT Alport trial.

EARLY PRO-TECT Alport is a phase III, multicenter, randomized, placebo-controlled, double-blind (patient, investigator and trial team http://www.clinicaltrials.gov/) clinical trial conducted in Germany according to the German Medicines Act (Arzneimittelgesetz). Up to 80 previously untreated AS patients with Alport stages 0 or I at screening (Table 1) will be randomized into once-daily treatment over 3 years with ramipril or placebo. Patients will be randomized (stratified by trial site) at a 1 : 1 ratio using permutated blocks (Figure 1). Upon disease progression (Table 2) during the treatment period, patients are unblinded, and ramipril treatment is initiated, if applicable. Approximately 40 AS patients will be treated open label with ramipril and will contribute to the safety database. This group includes previously treated as well as untreated patients who or whose parents/legal guardian refuses randomization because they request treatment.

### 2.2. Participants

Patient eligibility criteria are listed in Table 3. This trial includes patients with very early stages of AS (before proteinuria >0.3 g per day). As most pediatric nephrologists (>90%) today already treat AS children with proteinuria and/or renal impairment (unpublished data in [4]), not enough untreated AS patients at the stage of proteinuria would be available for a controlled clinical trial.

All newly diagnosed patients in Germany (or from outside Germany who are willing to travel to Germany) will be assessed for eligibility over the recruitment period of 2 years. Recruitment will be supported by the German and other national Alport patients' advocacy groups. The trial is supported by the Study Group of the German Society of Paediatric Nephrology.

### 2.3. Intervention

**Figure 2:**
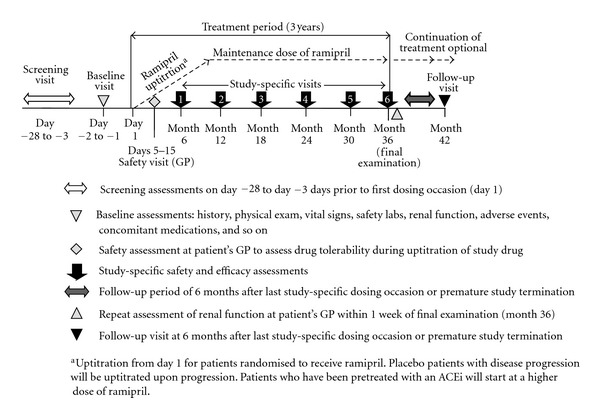
Study visits for the individual patient during the EARLY PRO-TECT Alport.

Over the treatment period of three years, randomised patients receive once-daily ramipril and placebo, respectively. For previously untreated patients, ramipril will initiated at 1 mg/m^2^ and uptitrated by 1 mg/m^2^ every 2 months over the first 10 months until the individual maximum tolerated dose (MTD) has been reached (Figure 2). 

For the open-label treatment group, patients who are pretreated with another ACEI before study entry will change treatment to ramipril within two weeks prior to the study-specific first dosing occasion. For patients under 18 years of age, the maximum daily dose of ramipril will be 6 mg/m^2^. From the age of 18 years, the maximum daily dose of ramipril will be 10 mg. After completion of the treatment period, treatment may be continued or initiated at the investigator's discretion and the patient's and/or parents'/guardians' wish. Ramipril was chosen as ACE inhibitor, because of the preclinical data in Alport mice [5] and clinical data in hypertensive children in the ESCAPE trial [8].

### 2.4. Sample Size

The sample size/power calculation is driven by the primary efficacy endpoint, that is, the time to progression of Alport syndrome to the “next disease level” (Table 2). The proportions of patients who remain in one disease level during the study period of 3 years were estimated to be approximately 50% on placebo and approximately 80% on ramipril; that is, 50% of patients on placebo and 20% of patients on ramipril are expected to progress in disease level. This corresponds to a hazard ratio of 0.322. With a two-sided test at *α* level of 0.05, equal allocation to both groups, and an assumed hazard ratio of 0.322, the numbers of events of “next disease level” required to achieve a power of 80% and 90% are 24 and 33 [10], respectively. A sample size of 80 patients will provide 28 events within a fixed 3-year observation period (intervention time) and will therefore give a power in excess of 80%. Dropouts are unlikely in this population, because older relatives in these families are often severely affected—increasing the high degree of compliance towards a potentially renal-protective therapy in patients and parents.

The total time on ramipril is expected to be about 270 person-years with the patients randomized to ramipril contributing by 120 person-years (40 patients for 3 years), the open-label use by another 120 person-years (40 patients for 3 years), and the patients randomized to placebo who receive ramipril upon disease progression contributing by 30 person-years (estimated 20 patients with disease progression after an average of 1.5 years). Assuming a Poisson distribution of the events, this total length of followup is sufficient to estimate an adverse event rate of 1 in 10 person-years with a precision of 20%.

### 2.5. Trial Duration

Overall trial duration is expected to be 5.5 years with a recruitment period of 2 years, a treatment period of 3 years, and a follow-up period of 0.5 years. Trial duration for the individual patient is 3.5 years. Study start (recruitment) is planned for March 2012.

### 2.6. Outcome Measures

During the treatment period, safety and efficacy assessments take place every six months. Safety will be assessed by physical examinations, laboratory tests, vital signs, concomitant medications, and the recording of adverse events. Potential side effects of ramipril in adult patients with indications other than Alport syndrome are listed in detail in the product specification file. Briefly, significant side effects in adults include hyperkalaemia, hypotension, angioedema, acute renal failure, cataract, and hepatitis. Major adverse drug reactions are expected in less than 1% of the study participants. The risk of acute renal failure exists in cases of serious exsiccation (e.g., as a result of serious diarrhoea). This correlation will be explained to patients and to their parents/legal guardian, respectively, in order to avoid the risk of acute renal failure. Efficacy will be assessed by laboratory tests (blood, urine), height, and weight. Additional serum and urine samples will be taken for the development of serum and urinary markers of renal disease progression (exploratory). A blood sample for DNA extraction may be taken if indicated to confirm the patient's Alport mutation (genetic testing is not part of this study). After completion of the treatment period, patients will be advised to undergo a hearing test and an eye examination.

### 2.7. Statistical Analysis

The primary efficacy endpoint “time to progression to the next disease level” for randomized patients will be assessed in 6-monthly intervals over the treatment period of 3 years. A proportional hazards' model for interval-censored data will be applied with effects for treatment, trial site, and proteinuria at start of therapy. If indicated, additional potentially confounding variables such as genotype and age will be added to the model. An estimate of the treatment effect is reported in terms of the hazard ratio with 95% confidence interval and *P* value testing the null hypothesis of no effect.

The primary safety endpoint is the “incidence of adverse drug events (ADE), for example, angioedema, acute renal failure, hyperkalemia, under ramipril treatment before disease progression compared to placebo before disease progression, for all randomized patients.” The incidence rates will be analyzed using a generalized linear model with log link (Poisson regression) and corrected, if necessary, for overdispersion due to interpatient heterogeneity. An estimate of the treatment effect is reported in terms of the rate ratio with 95% confidence interval and *P* value testing the null hypothesis of no effect.

The secondary efficacy endpoint “albuminuria after 3 years corrected for baseline albuminuria for patients randomized to receive ramipril compared to placebo” will be analyzed by means of an analysis of covariance (ANCOVA) with treatment, trial site, and proteinuria at start of therapy as factors and baseline albuminuria as covariate. The treatment effect estimate in terms of a mean difference will be reported with 95% confidence interval and *P* value testing the null hypothesis of no treatment effect. The *secondary safety endpoint* “incidence of ADEs during 3 years of treatment for patients randomized to receive ramipril compared to placebo” will be carried out in the same way as the primary safety analyses described above. Other secondary end points include eye involvement and hearing loss. Results will also be stratified by age groups. No interim analyses are planned.

### 2.8. Ethics Basics, Legislation and Guidelines, and Notification of the Authorities

The Ethics Committee approval concerning the suitability of the trial site and the qualifications of the investigators and conducting the trial was made by the leading Ethics Committee of the University Medical Center Göttingen, Germany (application number Az 11/6/11) in consulting all other participating German Ethics Committees. The clinical trial was also approved by the German competent authority, the Federal Institute for Drugs and Medical Devices (BfArM). Before starting the clinical trial, the state authorities in each federal state of Germany in which the clinical trial will be conducted must be notified.

The study is in adherence with the Declaration of Helsinki. This clinical trial will be conducted in accordance with the published principles of the guidelines for Good Clinical Practice (ICH-GCP) and applicable legislation (the German Medicines Act (AMG) and the GCP-V). All trial subjects enrolled are insured in accordance with § 40 German Medicines Act (AMG).

### 2.9. Data Quality Assurance and Dissemination

Each of the trial sites will be monitored to ensure the quality of the data collected. Monitoring on site will be performed in accordance with the trial-specific standard operating procedures (SOPs), the investigational plan, the guidelines for Good Clinical Practice (ICH-GCP), and local legislation (especially the German Medicines Act (AMG) and the GCP-V). An adapted monitoring concept will be used in accordance to a trial-specific risk assessment. Results will be published irrespective of outcome; written consent will be obtained from the patients/parents/legal guardian.

## 3. Discussion and Conclusion

ACE-inhibitor therapy in AS patients with proteinuria reflects the common off-label treatment [7]. However, long-term therapy with ACEIs in children may lead to long-term drug toxicities unknown in adults. This makes long-term off-label therapy an ethical distress for the pediatric nephrologist. Indications for initiation of treatment by pediatric nephrologists include hematuria alone (3%), microalbuminuria (27%), and overt proteinuria (70%) [4]. Children with AS and proteinuria are thus being treated in a way that prevents the objective assessment of the risks and benefits of therapy. A placebo-controlled clinical trial in children who have not yet developed overt proteinuria is therefore believed not to conflict with the current treatment practices of most pediatric nephrologists [4].

The primary aim of the EARLY PRO-TECT Alport trial is to assess drug safety and efficacy of ramipril treatment in oligosymptomatic pediatric AS patients in the setting of a prospective, placebo-controlled, GCP-conform clinical trial. An integrated translational program will help to define markers of renal disease progression that indicate risk, prognosis, disease activity, and response to therapy without the need for blood sampling or renal biopsy. The results are expected to be of relevance for almost all glomerular renal diseases. Finally, to maximize the available evidence, we will combine the treatment effect estimates from the randomized comparison and Alport registry data. This multimodal concept may serve as a model for other rare pediatric renal diseases, for which trials are similarly difficult to perform.

##  Authors' Contribution

O. Gross is the principal investigator and medical expert for the trial. T. Friede is the trial statistician and contributed to the design of the trial. R. Hilgers was the leading statistician during the early stages of the trial design development. Members of the Gesellschaft für Pädiatrische Nephrologie (GPN) performed scientific reviews of the protocol. A. Görlitz was responsible for the sample size calculations. R. Ahmed and K. Gavénis were responsible for regulatory affairs. U. Dürr was the medical writer for the protocol and associated documents.

##  Funding

This work is supported by the joint program on clinical trials by the German Research Council (DFG) and the German Federal Ministry of Education and Research (BMBF).

##  Conflict of Interests

None of the authors have significant competing financial, professional, or personal interests that might have influenced the performance or presentation of the work described in this paper.

## Figures and Tables

**Table 1:** Alport syndrome levels describing renal damage and loss of function.

Stage	Definition
0	Microhaematuria without microalbuminuria (usually at birth)
I	Microalbuminuria (30–300 mg albumin/gCrea)
II	Proteinuria >300 mg albumin/gCrea
III	>25% decline of normal renal function (creatinine clearance)
IV	End-stage renal failure (ESRF)

**Table 2:** Alport syndrome disease progression in this trial.

Progression	Definition
Stage 0 to I	Albuminuria >30 mg albumin/gCrea in combination with a 3-fold increase from baseline in albuminuria, confirmed within 2 weeks.
Stage I to II	(i) Twofold increase from baseline albuminuria, confirmed within 2 weeks
	(ii) Albuminuria >300 mg albumin/gCrea at a single assessment.

**Table 3:** Patient eligibility criteria.

	*Inclusion criteria*
	(i) Males or females of any ethnic origin aged between 24 months and <18 years at screening. Female patients of child-bearing potential test negative for pregnancy test and agree to use a reliable method of birth control during the study.
	(ii) Definitive diagnosis of Alport syndrome by kidney biopsy (in patient or other affected family members) and/or genetic testing (hemizygous males with X chromosomal Alport syndrome or homozygous males or females with autosomal recessive Alport syndrome) including screening for clinical diagnostic criteria (hematuria, family history of kidney disease, hearing loss, ocular changes).
	(iii) Alport syndrome stages 0 or I.
	(iv) Previously untreated with ACEIs (patients taking an ACEI for the treatment of Alport syndrome may be included in the open-label treatment arm).
	(v) Written informed consent.

	*Exclusion criteria*
	(i) Uncertain diagnosis or variants of Alport syndrome such as heterozygous carriers.
	(ii) Alport syndrome stages II, III, or IV.
	(iii) Known allergies or intolerances to ramipril or related compounds.
	(iv) Known contraindication for ACEI therapy (e.g., bilateral renal artery stenosis, prior history of angioedema, previous hyperkalemia, or previous acute prerenal renal failure).
	(v) Females with a positive pregnancy test or females who are lactating.
	(vi) Additional other chronic renal, liver, or cardiac diseases.
	(vii) No written informed consent.
